# Kinesiophobia Level in Fibromyalgia Patients and Its Relationship with Cognitive Functions, Anxiety, Depression, Disease Activity and Pain Level

**DOI:** 10.3390/healthcare14091137

**Published:** 2026-04-24

**Authors:** Köksal Sarihan, Ali İnaltekin, Onur Alp Yilmaz

**Affiliations:** 1 Department of Physical Medicine and Rehabilitation, Maçka Ömer Burhanoğlu Physical Therapy and Rehabilitation Hospital, Trabzon 61750, Türkiye; 2 Department of Psychiatry, Bilecik Şeyh Edebali University School of Medicine, Bilecik 11000, Türkiye; 3 Department of Psychiatry, Kocaeli City Hospital, Kocaeli 41060, Türkiye

**Keywords:** fibromyalgia, kinesiophobia, cognitive, pain, anxiety, depression

## Abstract

**Highlights:**

**What are the main findings?**

- The presence of increased kinesiophobia in FMS patients compared to the general population was determined.
- The level of kinesiophobia observed in patients with fibromyalgia syndrome (FMS) was significantly associated with both the severity of the disease and impairments in naming-related cognitive function.

**What are the implications of the main findings?**

- It is important to evaluate the kinesiophobia levels of FMS patients early.

**Abstract:**

**Background:** This cross-sectional study aimed to determine the status of kinesiophobia in patients with fibromyalgia syndrome (FMS) and to evaluate its relationship with cognitive functions, anxiety, depression, disease severity, and pain level. **Methods:** Fifty FMS patients (mean age 44.06 years; range 18 to 60 years) were included in the study. Fifty healthy participants (mean age 42.04 years; range 18 to 60 years) were included in the study as a control group. Participant recruitment for the study was conducted in a state hospital in Türkiye. Disease severity level in the FMS group was assessed using the Fibromyalgia Impact Questionnaire (FIQ). The Tampa Scale for Kinesiophobia (TSK) was administered to determine the participants’ kinesiophobia status. The Hospital Anxiety and Depression Scale (HADS) was used to assess the participants’ anxiety and depression status. The Montreal Cognitive Assessment (MoCA) test was administered to assess cognitive functions. **Results:** TSK, HADS-Anxiety, Visual Analog Scale (VAS), and FIQ scores were higher in the FMS group compared to the control group (*p* < 0.05). MoCA total scores, MoCA-Visuospatial/Executive, and MoCA-Attention scores were higher in the control group (*p* < 0.05). There was a weak positive correlation between TSK and FIQ scores in the FMS group (*p*: 0.02, r: 0.31). There was a weak negative correlation between TSK and MoCA-Naming scores in the FMS group (*p*: 0.02, r: −0.31). **Conclusions:** Increased kinesiophobia was found in FMS patients compared to the general population. It was determined that the level of kinesiophobia in FMS patients was related to disease severity and naming-related cognitive functions. Clinicians dealing with FMS should take this into particular consideration.

## 1. Introduction

Fibromyalgia syndrome (FMS) is a chronic musculoskeletal disorder characterized by chronic generalized body pain and morning stiffness, particularly in the neck, back, shoulder, and hip areas [1,2,3]. FMS prevalence peaks among women aged 40–60 [1,4]. The prevalence of FMS ranges from 1.3% to 8%, making it the second most common rheumatic disease after osteoarthritis [3,5]. In rheumatology clinics, this syndrome is detected in approximately 14% of patients [6]. The etiology of the disease remains unclear, but it is thought to involve alterations in nociceptor function, differences in pain perception, central sensitization, somatization, oxidative stress, neurotransmitters, and neuroendocrine disturbances [7,8].

Symptoms of fibromyalgia syndrome may include widespread pain, sensitivity to touch or pressure, fatigue, bowel problems, psychiatric disturbances, and sleep and memory problems [3,4,9]. The term “fibro fog” describes the cognitive dysfunction symptoms seen in FMS. These symptoms include decreased mental clarity, memory impairment, and reduced attention and focus [9,10]. Psychiatric disorders such as depression, anxiety, and post-traumatic stress disorder are common among patients with FMS [11,12]. Additionally, fear of movement may be observed in FMS patients [13,14].

Kinesiophobia can be expressed with various terms such as fear-avoidance beliefs, pain-related fears, and fear of movement [15,16]. Kinesiophobia is defined as an excessive, irrational, and debilitating fear of engaging in physical activities due to perceived vulnerability to painful injury or re-injury [17,18]. Kinesiophobia may emerge as a result of trauma but can also develop through observational and social learning [19,20].

The “fear-avoidance model” was developed to explain how kinesiophobia affects disability and chronicity in patients with musculoskeletal problems [21,22]. The fear-avoidance model is a theoretical framework that examines the relationship between chronic pain and cognitive–emotional factors [22]. The model states that negative appraisal of pain and its effects (pain catastrophes) is a precursor to pain-related fear. It suggests that this fear directs the patient’s attention to possible somatic threat signals and to avoidance/escape behavior [23]. In this model, also known as the “pain avoidance model,” there are two responses following an event such as an injury that causes pain. The first is a low-fear, confrontational response that leads to a recovery response. The second response creates a state in which movement is perceived as a threat, leading to the development of kinesiophobia, fear, and avoidance behavior [19,20]. Recent evidence highlights the mediating role of kinesiophobia in the relationship between pain intensity and reported disability [24].

Studies in patients with FMS have revealed increased levels of kinesiophobia compared to those in the general population [13,14,25]. A study found a relationship between kinesiophobia levels and pain scores and symptom duration in FMS patients [1]. Another study found a relationship between kinesiophobia scores and FMS disease severity [3]. Cognitive processes such as an overfocus on pain, misconceptions about movement, and low self-efficacy may lead to misinterpretation of pain, avoidance of movement, and, consequently, kinesiophobia. To our knowledge, there are no studies in the literature examining the relationship between kinesiophobia and cognitive functions in patients with fibromyalgia. This cross-sectional study aimed to determine the status of kinesiophobia in patients with FMS and to evaluate its relationship with cognitive functions, depression, anxiety, pain level, and disease severity.

**The study’s hypotheses:**

**H1.**  *Kinesiophobia is more common in FMS patients than in the general population.*

**H2.**  *There is a relationship between cognitive functions and kinesiophobia in patients with FMS.*

**H3.**  *There is a relationship between disease activity, pain, anxiety, depression, and kinesiophobia in FMS patients.*

## 2. Materials and Methods

### 2.1. Study Design

Participant data for this study were collected between December 2020 and September 2021 at a physical medicine and rehabilitation outpatient clinic in a state hospital in Türkiye. This study is a cross-sectional study.

### 2.2. Participants

Fifty consecutive patients with FMS (mean age 44.06 years; age range 18 to 60 years) who met the American College of Rheumatology’s 2016 revised diagnostic criteria and were followed up by a physical medicine and rehabilitation specialist were included in the study [26]. Fifty healthy volunteers (mean age 42.04 years; age range 18 to 60 years), matched for age, gender, and education level, who presented to the hospital for screening, were included as the control group. The inclusion criteria for the study were as follows: having an FMS diagnosis (for the FMS group), providing written informed consent, and being competent to understand the items on the administered scale. Patients with inflammatory rheumatic diseases, those who had undergone major musculoskeletal surgery, those diagnosed with cancer, those with vestibular problems, pregnant or breastfeeding women, those with psychiatric diseases, morbidly obese patients, and those with serious neurological disorders were not included in the study.

### 2.3. Outcome Measures

Participant information forms were completed to collect demographic and clinical data, including age, gender, educational duration, and the presence of comorbid chronic conditions.

Participants’ pain levels were assessed using the Visual Analog Scale (VAS) [27]. Participants were provided with information about a scale ranging from 0 to 10. They were asked to rate their pain on a scale of 0 to 10, with 0 points indicating no pain and 10 points indicating the most severe pain. Participants were asked to describe their pain intensity accordingly.

Disease severity was determined using the Fibromyalgia Impact Questionnaire (FIQ). This questionnaire includes 10 different subscales that evaluate well-being, physical disability, lost workdays, and the number of days the participant is able to work. The total score ranges from 0 to 100, with higher scores indicating greater impact. The Turkish validity and reliability of FIQ were established by Sarmer et al. [28].

To assess kinesiophobia status, participants were administered the Tampa Scale for Kinesiophobia (TSK). This scale consists of 17 items. The score for each question ranges from 1 to 4. The total on the scale ranges from 17 to 68. A higher score is associated with higher kinesiophobia. According to this scale, scores below 37 indicate a “low level of fear of movement,” whereas scores above 37 indicate a “high level of fear of movement”. The Turkish validity and reliability of TSK were established by Yılmaz T et al. [29].

The anxiety and depression levels of the participants were assessed using the Hospital Anxiety Depression Scale (HADS). This scale was developed for screening depression and anxiety symptoms in individuals with physical illness. The scale consists of a total of 14 items, 7 for depression and 7 for anxiety symptoms. Responses are evaluated on a four-point Likert scale, ranging from 0 to 3. Higher scores indicate higher levels of anxiety and depression. The Turkish validity and reliability of this scale were established by Aydemir Ö et al. [30].

Participants were administered the Montreal Cognitive Assessment (MoCA) to assess cognitive function. This scale was developed to differentiate healthy individuals from those with mild cognitive impairment. The scale includes questions that evaluate executive functions, memory, visual–spatial skills, language, attention and concentration, calculation and orientation, and abstract thinking. An additional point is awarded to participants with 12 years of education or less. The maximum score on the scale is 30, and a threshold of 21 is used (scores of 20 or lower indicate cognitive impairment). The Turkish validity and reliability study of this scale was conducted by Selekler K et al. [31] and Özdilek B et al. [32].

All assessments, including fibromyalgia disease severity, pain intensity, and kinesiophobia, were conducted by the same investigator. Similarly, evaluations of depression, anxiety, and cognitive function were performed by the same assessor.

### 2.4. Study Ethics

Approval was received from the ethics committee of a training and research hospital (Decision No: BEAK KAEK 2020/23-226). The study was conducted in accordance with the principles of the Declaration of Helsinki. All patients signed a written informed consent form.

### 2.5. Statistical Analysis

The SPSS Version 23.0 software package (IBM, Chicago, IL, USA) was used for statistical calculations. Descriptive statistics were used to assess whether numerical variables followed a normal distribution, employing the Shapiro–Wilk test. The *t*-test was used to compare numerical variables, and the chi-square test was used to compare categorical data. Pearson’s correlation analysis was used. Categorical data are expressed as numbers (percentages), and numerical data are expressed as means ± standard deviations (SD). Effect sizes were calculated using Cohen’s d to assess the magnitude of group differences. Cohen’s d values of 0.20, 0.50, and 0.80 were interpreted as small, medium, and large effect sizes, respectively. *p* < 0.05 was considered statistically significant.

To conduct this study, data from a previous study evaluating kinesiophobia in patients with FMS were used. According to the data from this study, using mean TSK scores of 42.0 ± 7.6 for the patient group and 37.2 for the control group, it was calculated that at least 40 participants were required in each group, with 95% confidence and 80% power [3]. The G-Power 3.1 program was used for this calculation. Considering potential data-collection issues and to ensure an adequate sample size, it was planned to include 50 participants in each group.

## 3. Results

There were no differences between the two groups in terms of sociodemographic characteristics and the presence of additional chronic diseases (*p* > 0.05) (Table 1).

VAS scores, TSK scores, HADS-Anxiety scores, and FIQ scores were higher in the FMS group than in the control group (*p* < 0.01, *p* = 0.01, *p* < 0.01, and *p* < 0.01, respectively). This result represents a medium effect size. MoCA-Total, MoCA-Visuospatial/Executive, and MoCA-Attention scores were also higher in the control group than in the FMS group (*p* = 0.04, *p* < 0.01, and *p* = 0.01, respectively). There were no differences in the other clinical parameters evaluated (*p* > 0.05) (Table 2).

In the FMS group, there was a weak positive correlation between TSK scores and FIQ scores (*p* = 0.02, r = 0.31). There was no significant relationship between TSK scores and the other evaluated parameters (Table 3).

In the FMS group, there was a weak negative correlation between TSK scores and MoCA-Naming scores (*p* = 0.02, r = −0.31). There was no significant relationship between TSK scores and the other evaluated parameters (Table 4).

## 4. Discussion

Based on the results of our study, the FMS group had higher levels of kinesiophobia than the healthy population. We also identified a weak positive correlation between kinesiophobia levels and fibromyalgia disease severity in the FMS group. We found a weak negative correlation between kinesiophobia levels and MoCA-Naming scores in the FMS group.

Kinesiophobia is important due to its impact on physical activity levels, activities of daily living, and overall quality of life [13,17,21,33]. Previous studies have found higher levels of kinesiophobia in FMS patients compared to healthy populations [13,14,25]. In a study of patients undergoing lumbar stabilization, the minimum clinically important difference for TSK was approximately 6 [34]. A study of patients with chronic low back pain found that a difference of at least 4 points on the TSK was required to be certain of a change in fear of movement [35]. Our study found that the FMS group had higher kinesiophobia scores than the control group. In our study, the difference in TSK scores between the control and FMS groups exceeds 3. In our study, Cohen’s d was calculated for the TSK between the FMS and control groups, yielding a value of 0.48. This result represents a medium effect size. Our study results, consistent with the literature, revealed that patients with FMS have higher kinesiophobia scores. To determine whether the difference in TSK scores between FMS patients is clinically significant, the minimum clinically significant difference must be determined.

Various levels of cognitive impairment can be detected in FMS patients, with common reports of deficits in attention, planning, memory, executive functions, and processing speed [36,37,38]. In our study, we found that FMS patients may have lower MoCA-Total, Visuospatial/Executive, and Attention scores than the general population. Kinesiophobia is primarily assumed to occur due to factors that induce changes in cognitive structuring, such as fear associated with pain and pain catastrophizing [15,39]. Therefore, a relationship between cognitive impairments and kinesiophobia in patients with FMS would be expected. In our study, we identified a weak negative correlation between naming sub-scores of cognitive functions and kinesiophobia scores. Our study found a relationship between kinesiophobia and naming; however, the literature on this topic is limited. This study has laid the groundwork for future research. Further studies with more detailed domain analysis of cognitive functions are needed.

In one study, an association between depression scores and kinesiophobia scores was found in FMS patients [40]. In another study, a relationship between depression scores and kinesiophobia scores in FMS patients was also observed [41]. Another study detected a relationship between anxiety scores and kinesiophobia [41]. However, in our study, unlike the literature, we did not find a relationship between depression scores, anxiety scores, and kinesiophobia scores. This may be due to the frequent occurrence of other psychiatric conditions in patients with FMS that may be associated with kinesiophobia and may have a statistically confounding effect.

In a study conducted on participants with chronic musculoskeletal pain, it was found that pain scores and kinesiophobia levels were related [17]. In one study, a positive relationship was found between kinesiophobia scores and pain scores in FMS patients [40]. In another study, while a weak positive correlation was found between kinesiophobia and VAS-Night pain in patients with FMS, no relationship was found between kinesiophobia and VAS-Rest pain or VAS-Motion pain [19]. In one study, a positive correlation was found between kinesiophobia and disease severity in FMS patients [42]. In another study, no relationship was found between disease severity and kinesiophobia in FMS patients [40]. In our study, there was no relationship between kinesiophobia levels and pain scores in FMS patients, but there was a positive relationship with disease severity. The association between pain and kinesiophobia levels in our study may not have been detected because of confounding factors such as comorbid fatigue, vertigo, vestibular system disorders, and panic disorder [42]. Because some of these were assessed within the Fibromyalgia Impact Questionnaire, we may have been able to identify a relationship between disease severity and kinesiophobia.

One limitation of this study is that participants’ physical activity habits and activity levels were not evaluated. Secondly, causality may not have been fully established due to the study’s cross-sectional design. Thirdly, the gender distribution is not homogeneous. Fourthly, the MoCA test is used for screening purposes in cognitive function assessment. This test does not offer detailed domain analysis. In this respect, the results of our study should be interpreted with caution. Fifthly, we did not assess medication use status. Finally, since correlation analyses are both exploratory and hypothesis-driven, no formal corrections for multiple comparisons were applied. However, we acknowledge that the risk of type I error associated with multiple testing is increased. The value of this study lies in the fact that, to our knowledge, it is the first to investigate the relationship between cognitive functions and kinesiophobia levels in patients with FMS.

## 5. Conclusions

In conclusion, increased kinesiophobia was observed in patients with FMS compared to the general population. Furthermore, the level of kinesiophobia in patients with FMS may be related to disease severity and naming function. It is important to evaluate the kinesiophobia levels of patients with FMS at an early stage. If kinesiophobia is present, the development of preventive strategies may provide additional benefits in the treatment plan. It is recommended that future studies examine the relationship between kinesiophobia and panic disorders, vertigo, fatigue, and cognitive functions in patients with FMS.

## Figures and Tables

**Table 1:** Additional chronic disease status and sociodemographic characteristics of the FMS group and control group.

	FMS Group (*n*: 50)	Control Group (*n*: 50)	*p*
Age (Year) (Mean ± SD)	44.06 ± 9.40	42.04 ± 11.65	0.24
Gender, Number (%)			
Male	9 (18)	5 (10)	
Female	41 (82)	45 (90)	0.24
Education duration (Year) (Mean ± SD)	8.72 ± 3.37	9.40 ± 3.38	0.31
Presence of Additional Chronic Disease (n (%))			
Yes	28 (56)	26 (52)	0.68
No	22 (44)	24 (48)	

FMS: fibromyalgia syndrome.

**Table 2:** Clinical characteristics and scale scores of the FMS group and control group.

	FMS Group (*n*: 50)	Control Group (*n*: 50)	*p*	Cohen’s d
TSK (Mean ± SD)	41.62 ± 7.19	38.50 ± 5.76	0.01	0.48
MoCA-Total (Mean ± SD)	20.14 ± 5.54	22.32 ± 5.00	0.04	0.41
Visuospatial/Executive (Mean ± SD)	2.82 ± 1.38	3.56 ± 1.18	<0.01	0.58
Naming (Mean ± SD)	2.72 ± 0.45	2.84 ± 0.47	0.19	
Attention (Mean ± SD)	3.50 ± 1.75	4.42 ± 1.80	0.01	0.52
Language (Mean ± SD)	1.46 ± 0.99	1.58 ± 0.99	0.54	
Abstraction (Mean ± SD)	1.08 ± 0.90	1.12 ± 0.77	0.81	
Delayed Recall (Mean ± SD)	2.96 ± 1.70	3.08 ± 1.66	0.72	
Orientation (Mean ± SD)	5.50 ± 1.04	5.70 ± 0.68	0.25	
MoCA-Presence of Cognitive Impairment (n (%))				
Yes	22 (44)	15 (30)		
No	28 (56)	35 (70)	0.14	
HADS-Anxiety (Mean ± SD)	9.38 ± 4.26	7.24 ± 3.30	<0.01	0.56
HADS-Depression (Mean ± SD)	8.02 ± 4.29	6.82 ± 4.06	0.15	
VAS (Pain) (Mean ± SD)	7.12 ± 2.29	5.54 ± 2.56	<0.01	0.65
FIQ (Mean ± SD)	55.00 ± 18.20	42.32 ± 18.72	<0.01	0.69

FMS: fibromyalgia syndrome; TSK: Tampa Scale for Kinesiophobia; MoCA: Montreal Cognitive Assessment; HADS: Hospital Anxiety and Depression Scale; VAS: Visual Analog Scale; FIQ: Fibromyalgia Impact Questionnaire.

**Table 3:** Relationship between TSK scores and clinical characteristics in the FMS group (*n*: 50).

		FIQ	HADS-Anxiety	HADS-Depression	Education Duration	VAS	Age
TSK	r	0.31	−0.02	0.13	0.05	0.08	−0.09
	*p*	0.02	0.87	0.33	0.72	0.57	0.53

Pearson correlation analysis was used. Correlation is significant at the 0.05 level (2-tailed). TSK: Tampa Scale for Kinesiophobia; FMS: fibromyalgia syndrome; FIQ: Fibromyalgia Impact Questionnaire; HAD: Hospital Anxiety and Depression Scale; VAS: Visual Analog Scale.

**Table 4:** Relationship between TSK scores and MoCA-Total and subscale scores in the FMS group (*n*: 50).

		MoCA -Total	Visuospatial/Executive	Naming	Attention	Language	Abstraction	Delayed Recall	Orientation
TSK	r	<0.01	0.08	−0.31	0.18	0.09	0.27	0.20	0.27
	*p*	0.97	0.56	0.02	0.20	0.51	0.05	0.16	0.05

Pearson’s correlation analysis was used. Correlation is significant at the 0.05 level (2-tailed). TSK: Tampa Scale for Kinesiophobia; MoCA: Montreal Cognitive Assessment; FMS: fibromyalgia syndrome.

## Data Availability

The datasets for this study are not publicly available due to patient confidentiality, but they are available from the corresponding author upon reasonable request.
